# Corylin Attenuates CCl_4_-Induced Liver Fibrosis in Mice by Regulating the GAS6/AXL Signaling Pathway in Hepatic Stellate Cells

**DOI:** 10.3390/ijms242316936

**Published:** 2023-11-29

**Authors:** Chin-Chuan Chen, Chi-Yuan Chen, Chau-Ting Yeh, Yi-Tsen Liu, Yann-Lii Leu, Wen-Yu Chuang, Yin-Hwa Shih, Li-Fang Chou, Tzong-Ming Shieh, Tong-Hong Wang

**Affiliations:** 1 Biobank, Chang Gung Memorial Hospital, Tao-Yuan 33305, Taiwan; chinchuan@mail.cgu.edu.tw (C.-C.C.); d49417002@gmail.com (C.-Y.C.); crea456m@gmail.com (Y.-T.L.); ylleu@mail.cgu.edu.tw (Y.-L.L.); 2 Graduate Institute of Natural Products, Chang Gung University, Tao-Yuan 33303, Taiwan; 3 Graduate Institute of Health Industry and Technology, Research Center for Chinese Herbal Medicine and Research Center for Food and Cosmetic Safety, Chang Gung University of Science and Technology, Tao-Yuan 33303, Taiwan; 4 Liver Research Center, Department of Hepato-Gastroenterology, Chang Gung Memorial Hospital, Tao-Yuan 33305, Taiwan; chautingy@gmail.com; 5 Department of Anatomic Pathology, Chang Gung Memorial Hospital, Tao-Yuan 33305, Taiwan; s12126@cgmh.org.tw; 6 College of Medicine, Chang Gung University, Tao-Yuan 33303, Taiwan; 7 Department of Healthcare Administration, Asia University, Taichung 41354, Taiwan; evashih@gm.asia.edu.tw; 8 Kidney Research Center, Chang Gung Memorial Hospital, Tao-Yuan 33305, Taiwan; d928209@gmail.com; 9 School of Dentistry, China Medical University, Taichung 40402, Taiwan

**Keywords:** corylin, anti-inflammation, liver fibrosis, hepatic stellate cell, growth arrest-specific gene 6/AXL signaling pathway

## Abstract

Liver fibrosis is reversible when treated in its early stages and when liver inflammatory factors are inhibited. Limited studies have investigated the therapeutic effects of corylin, a flavonoid extracted from *Psoralea corylifolia* L. (Fabaceae), on liver fibrosis. Therefore, we evaluated the anti-inflammatory activity of corylin and investigated its efficacy and mechanism of action in ameliorating liver fibrosis. Corylin significantly inhibited inflammatory responses by inhibiting the activation of mitogen-activated protein kinase signaling pathways and the expression of interleukin (IL)-1β, IL-6, and tumor necrosis factor-alpha in human THP-1 and mouse RAW264.7 macrophages. Furthermore, corylin inhibited the expression of growth arrest-specific gene 6 in human hepatic stellate cells (HSCs) and the activation of the downstream phosphoinositide 3-kinase/protein kinase B pathway. This inhibited the activation of HSCs and the expression of extracellular matrix proteins, including α-smooth muscle actin and type I collagen. Additionally, corylin induced caspase 9 and caspase 3 activation, which promoted apoptosis in HSCs. Moreover, in vivo experiments confirmed the regulatory effects of corylin on these proteins, and corylin alleviated the symptoms of carbon tetrachloride-induced liver fibrosis in mice. These findings revealed that corylin has anti-inflammatory activity and inhibits HSC activation; thus, it presents as a potential adjuvant in the treatment of liver fibrosis.

## 1. Introduction

Liver fibrosis, caused by viral or metabolic chronic liver diseases, is a major challenge of global health [1,2]. In Taiwan, approximately 60% of patients with liver cancer have previously suffered from viral hepatitis B and C infections [3,4,5]. Chronic hepatitis causes repeated liver inflammation and activates hepatic stellate cells (HSCs) to secrete collagen for tissue repair. Consequently, the extracellular matrix (ECM) accumulates during repeated inflammation and repair, leading to liver fibrosis and liver cirrhosis. Patients with cirrhosis are 60–250 times more likely to develop liver cancer than those without liver disease [6].

Liver fibrosis can be reversed by administering treatment at an early stage and inhibiting the factors that cause liver inflammation [7]. Current treatment options for liver fibrosis can be classified based on three strategies: the inhibition of liver inflammation, the inhibition of HSC activation, and the acceleration of ECM breakdown. In the clinical setting, the administration of antiviral drugs alone, such as entecavir or lamivudine, or interferon (IFN) alone, may help ameliorate liver fibrosis caused by viral hepatitis; however, these treatments show low efficacy [8,9]. The treatment outcomes of liver fibrosis can be effectively improved if an appropriate adjuvant is administered to suppress inflammation or inhibit HSC activation. However, no clinically effective drugs are currently available for treating liver fibrosis with low side effects; thus, continuous research and development are required.

HSCs play a key role in the progression of liver fibrosis. When liver tissues are injured or stimulated by oxidative stress or inflammatory cytokines, HSCs are activated followed by proliferation and transformation into fibrogenic cells, thereby synthesizing large amounts of ECM. HSC activation is regulated by various pathways, with the growth arrest-specific 6 (GAS6)/AXL receptor tyrosine kinase (AXL) being a key regulatory pathway [10,11,12]. AXL, a member of the TYRO-AXL-MER (TAM) receptor tyrosine kinase (RTK) family, is mainly expressed in neural, vascular, immune, and stellate cells and is involved in the regulation of cellular physiological processes, such as growth, survival, differentiation, adhesion, and migration [13]. The binding of AXL to its ligand protein, GAS6, initiates autophosphorylation, which further activates the downstream phosphoinositide 3-kinase/protein kinase B (PI3K/AKT), rat sarcoma/rapidly accelerated fibrosarcoma kinase/mitogen-activated protein kinase (MAPK) kinase/extracellular signal-regulated kinase (RAS/RAF/MEK/ERK), and wingless-related integration site (Wnt) signaling pathways, thereby promoting cell growth, migration, and angiogenesis and inhibiting apoptosis [14]. The GAS6/AXL signaling pathway regulates HSC proliferation and activation, which play an important role in liver fibrosis development. The treatment of carbon tetrachloride (CCl_4_)-exposed mice with an AXL inhibitor effectively alleviates fibrosis symptoms [10]. Therefore, at present, TAM receptors, including AXL, are considered as key targets for treating liver fibrosis [15,16].

Natural products contain diverse pharmacophores and highly complex stereochemistry, and most of them have low physiological toxicity. Therefore, natural products have always represented important sources for new drug development [17,18]. Compounds, such as paclitaxel, curcumin, camptothecin, and their derivatives, have been used to treat various cancers, such as breast cancer, lung cancer, colorectal cancer, and melanoma, as they significantly prolong patient survival time [19,20,21,22,23]. Other natural compounds, such as resveratrol, metformin, magnolol, sulforaphane, and diallyl disulfide, exhibit anti-inflammatory activity and have the potential to be used in the treatment of inflammatory diseases [24,25,26,27,28].

*Psoralea corylifolia* L. (cullen corylifolium; Fabaceae) is an herb widely used for treating bacterial infections, inflammation, and cancers in many Asian countries [29,30,31]. Its polyphenolic extracts, such as psoralen, isopsoralen, and psoralidin; flavonoid extracts, such as bavachin, isobavachalcone, and neobavaisoflavone; and the phenolic extract backuchiol have all been identified as biologically active with different therapeutic effects [32]. Corylin, a flavonoid isolated from the fruits of *P. corylifolia* L., exerts an anti-inflammatory effect by inhibiting the expression of inducible nitric oxide synthase and cyclooxygenase which is increased during bacterial infections [33,34]. In addition, the antioxidant, anti-aging, and anti-tumor activities of corylin have also been reported recently [35,36,37,38], and have also shown the therapeutic potential of corylin in hyperlipidemia, insulin resistance, atherosclerosis, hepatocellular carcinoma, and neurological diseases [39,40,41]. We previously showed that corylin ameliorates obesity by activating adipocyte browning and reduces hepatic steatosis and hepatic fibrosis in high-fat diet (HFD)-fed mice [38]. However, the molecular mechanism of corylin’s anti-inflammation and anti-hepatic fibrosis effects have not yet been fully clarified. Therefore, in this study, we investigated the anti-inflammatory and therapeutic effects of corylin on liver fibrosis and further clarified its downstream regulatory mechanisms. Our findings showed that corylin has anti-inflammatory activity and inhibits HSC activation; thus, it can be used as a potential adjuvant in the treatment of liver fibrosis.

## 2. Results

### 2.1. Corylin Treatment Suppressed Lipopolysaccharide-Induced Pro-Inflammatory Cytokine Production in THP-1 and RAW264.7 Cells

**Figure 1:**
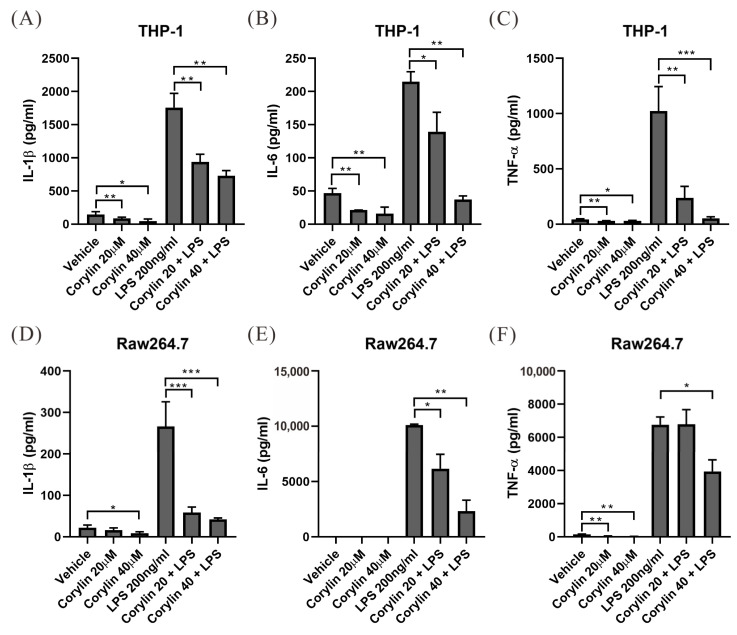
Corylin significantly inhibits IL-1β, IL-6, and TNF-α expression in THP-1 and RAW264.7 cells. Expression of pro-inflammatory cytokines, according to enzyme-linked immunosorbent assays, in (**A**–**C**) THP-1 and (**D**–**F**) RAW264.7 cells that were treated with different concentrations of corylin or vehicle for 2 h and then treated with 200 ng/mL LPS to induce an inflammatory response for 24 h. All data are expressed as the mean ± standard deviations of three independent experiments. *p* < 0.05 (*), *p* < 0.01 (**), *p* < 0.001 (***). LPS, lipopolysaccharide; IL-1β, interleukin 1 beta; TNF-α, tumor necrosis factor alpha; TGF-β, transforming growth factor beta.

To determine whether corylin exhibits anti-inflammatory activity, we treated human monocyte THP-1 cells and RAW 264.7 mouse macrophage cells with different concentrations of corylin for 2 h followed by lipopolysaccharide (LPS) treatment for 24 h to induce an inflammatory response. The culture media were collected to perform an enzyme-linked immunosorbent assay (ELISA) to analyze the expression of pro-inflammatory cytokines. LPS treatment significantly increased the expression of cytokines, such as interleukin (IL)-1β, IL-6, and tumor necrosis factor alpha (TNF-α), in THP-1 and RAW264.7 cells. The expression of these pro-inflammatory cytokines was significantly reduced in corylin-treated cells compared to that in the control group (dimethyl sulfoxide (DMSO)-treated), indicating that corylin exhibited anti-inflammatory activity and inhibited the expression of pro-inflammatory cytokines (Figure 1).

### 2.2. Corylin Treatment Inhibited the Activation of MAPK Signaling Pathways in LPS-Stimulated THP1 and RAW264.7 Cells

**Figure 2:**
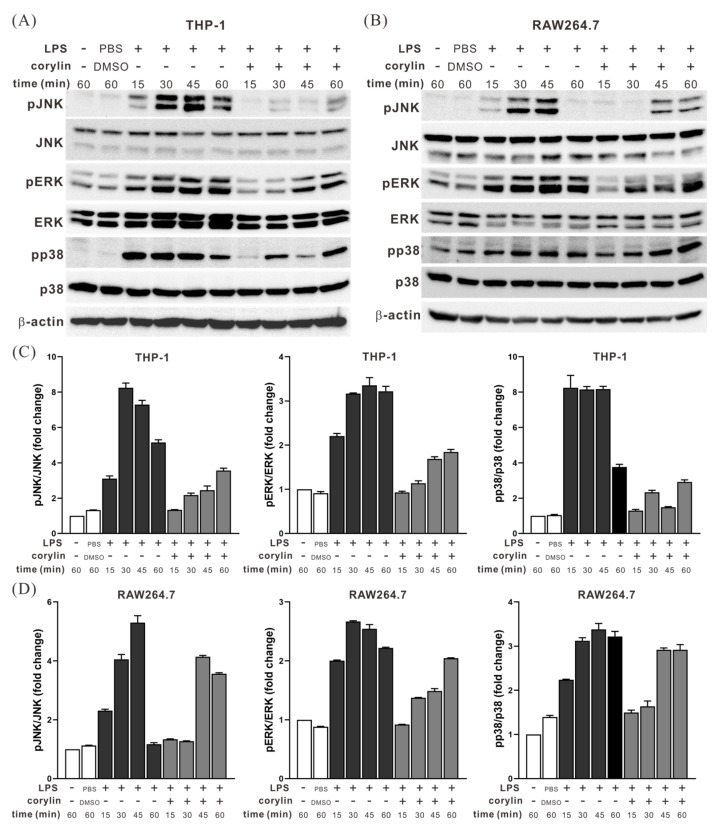
Corylin inhibits mitogen-activated protein kinase activation in THP-1 and RAW264.7 cells. (**A**) THP-1 cells and (**B**) RAW264.7 cells stimulated with 200 ng/mL LPS and 40 mM corylin for the indicated time period, and the activities of JNK, ERK, and p38 examined using Western blot analysis with phosphospecific antibodies are shown. (**C**,**D**) The total protein levels of JNK, ERK, and p38 were measured, and quantitative results are shown. LPS, lipopolysaccharide; JNK, c-Jun N-terminal kinase; ERK, extracellular signal-regulated kinase; p-JNK, phosphorylated JNK; IL-1β, interleukin 1 beta; TNF-α, tumor necrosis factor alpha; TGF-β, transforming growth factor beta.

To further determine whether the anti-inflammatory effect of corylin was associated with MAPK signaling pathways, THP-1 and RAW264.7 cells were pre-treated with corylin and stimulated with LPS. Subsequently, the phosphorylation levels of c-Jun N-terminal kinase, ERK, and p38 proteins were analyzed using Western blotting. LPS treatment activated the aforementioned MAPKs, which subsequently upregulated pro-inflammatory cytokines. However, in corylin-treated cells, the activation of these kinases was significantly inhibited (Figure 2), leading to the decreased expression of pro-inflammatory cytokines. Therefore, the anti-inflammatory activity of corylin was mediated by blocking the activation of MAPK signaling pathways.

### 2.3. Corylin Treatment Alleviated the Symptoms of CCl_4_-Induced Liver Fibrosis in Mice

**Figure 3:**
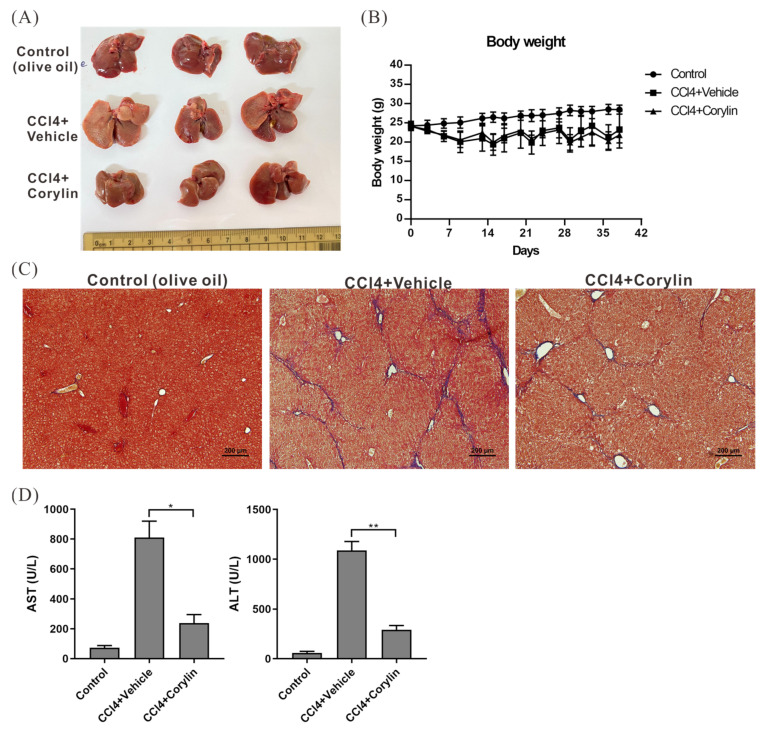
Effect of corylin on liver fibrosis in mice induced by CCl_4_ treatment. (**A**) Mice livers treated with CCl_4_, CCl_4_ + corylin, or olive oil (NC), as described in Section 4, are shown. Corylin treatment reduces liver fibrosis symptoms in CCl_4_-exposed mice six weeks after drug administration. (**B**) Body weights measured every three days after CCl_4_ injection are shown. (**C**) Masson’s trichrome staining reveals the effects of corylin on CCl_4_-induced liver fibrosis. (**D**) Effect of corylin on serum AST and ALT levels in mice. *p* < 0.05 (*), *p* < 0.01 (**). CCl_4_, carbon tetrachloride; AST, aspartate aminotransferase; ALT, alanine transaminase.

To confirm the anti-inflammatory activity of corylin and its efficacy in treating liver fibrosis in vivo, BALB/c mice were intraperitoneally injected with CCl_4_ (0.5 μL/g body weight) twice a week for six weeks to induce liver fibrosis. Further, the mice were intraperitoneally injected with/without corylin (30 mg/kg of body weight). After six weeks of CCl_4_ treatment, the mice exhibited significant fibrosis of the liver tissue, whereas liver fibrosis in corylin-treated mice was significantly alleviated compared with that in mice without corylin treatment (Figure 3A–C). Serological analysis also revealed that liver function indicator levels, including aspartate aminotransferase (AST) and alanine transaminase (ALT), in CCl_4_-treated mice were 8–10-fold higher than those in untreated mice, indicating that their liver tissues were in an inflammatory and injured state. In contrast, liver function indicator levels in corylin-treated mice were significantly reduced, indicating that corylin effectively inhibited liver tissue inflammation and injury caused by CCl_4_ (Figure 3D).

### 2.4. Corylin Treatment Inhibited HSC Activation

**Figure 4:**
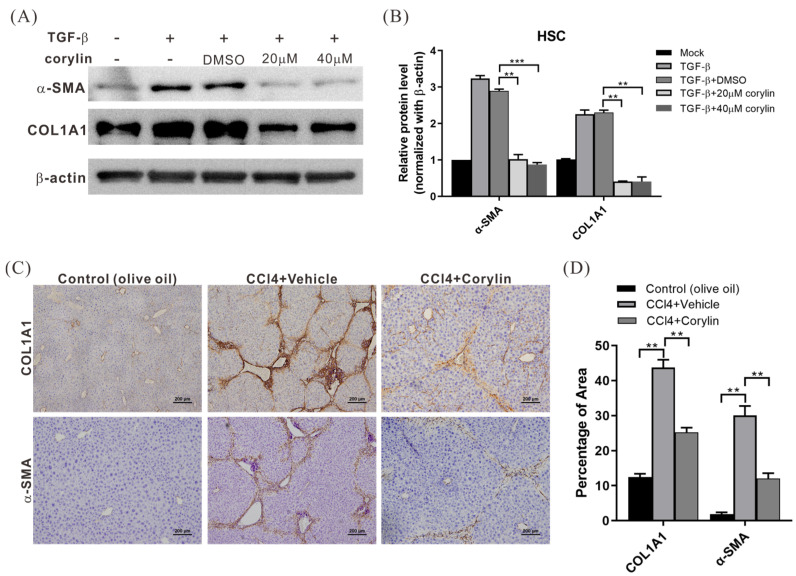
Corylin inhibits HSC activation. (**A**) Western blot analysis of human HSCs HHSteC cells treated with corylin or vehicle for 2 h, followed by TGF-β (4 ng/mL) treatment for 24 h to stimulate cell activation, to analyze α-SMA and COL1A1 protein expression, and to determine the effects of corylin on HSC activation. Quantitative results are shown in (**B**). All data are expressed as the mean ± standard deviations of three independent experiments. *p* < 0.01 (**), *p* < 0.001 (***). (**C**) Immunohistochemical staining representing the effects of corylin on COL1A1 and α-SMA expression in mouse livers. Quantitative results are shown in (**D**). *p* < 0.01 (**). HSCs, hepatic stellate cells; COL1A1, collagen 1A; α-SMA, smooth muscle-actin; TGF-β, transforming growth factor beta; DMSO, dimethyl sulfoxide.

Liver fibrosis is caused by the excessive accumulation of ECM proteins, such as collagen, which are secreted by activated HSCs. To determine whether corylin inhibits HSC activation, HHSteC cells were treated with corylin or a vehicle for 2 h, followed by transforming growth factor-β (TGF-β) treatment for 24 h to stimulate cell activation. Western blotting was performed to analyze the expression of alpha-smooth muscle actin (α-SMA) and collagen 1A to determine the effects of corylin on HSC activation. The expression of α-SMA and collagen 1A decreased significantly in HHSteC cells treated with corylin compared with that in the control group, indicating that corylin inhibited HSC activation (Figure 4A,B). In addition, immunohistochemical staining also showed that the expression of α-SMA and collagen 1A was significantly reduced in the tissues of corylin-treated mice compared to that in mice in the control group (Figure 4C,D). Therefore, corylin retards the progression of liver fibrosis in mice by inhibiting HSC activation.

### 2.5. Corylin Treatment Inhibited HSC Activation by Suppressing GAS6 Expression and Downstream PI3K/AKT Pathway Activation

**Figure 5:**
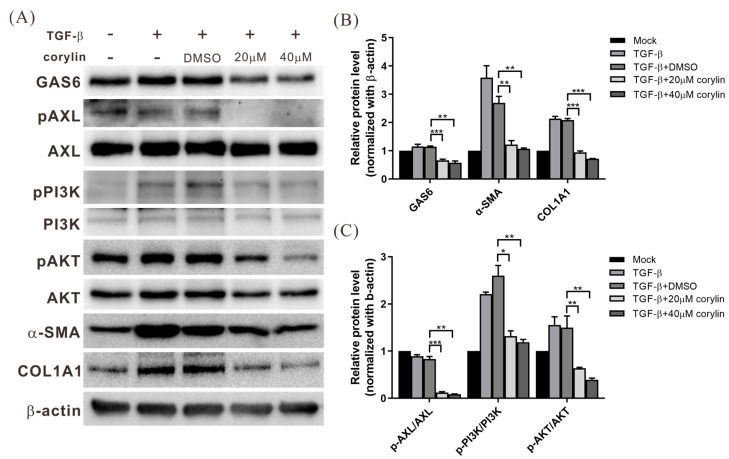
Corylin inhibits HSC activation by suppressing GAS6 expression and downstream PI3K/AKT signaling. (**A**) Western blotting results of cell lysates of HHSteC cells treated with corylin or DMSO for 2 h, followed by TGF-β (4 ng/mL) treatment for 24 h to stimulate cell activation and to determine the effects of corylin on the GAS6/AXL signaling pathway. β-Actin is the internal control. Quantitative results are shown in (**B**,**C**). All data are expressed as the mean ± standard deviations of three independent experiments. *p* < 0.05 (*), *p* < 0.01 (**), *p* < 0.001 (***). HSCs, hepatic stellate cells; GAS6, growth arrest-specific gene 6; PI3K/AKT, phosphoinositide 3-kinase/protein kinase B; p-PI3K, phosphorylated PI3K; DMSO, dimethyl sulfoxide; TGF-β, transforming growth factor beta; COL1A1, collagen 1A; α-SMA, smooth muscle-actin.

The GAS6/AXL signaling pathway is important in regulating HSC activation [10]. To determine the effects of corylin on the expression of GAS6 and AXL and their downstream regulatory pathways, HHSteC cells were treated with corylin or vehicle for 2 h, followed by TGF-β treatment for 24 h to stimulate cell activation. Western blotting was performed to analyze the effects of corylin on the GAS6/AXL signaling pathway. GAS6 expression was significantly reduced in corylin-treated cells compared to that in the control group (DMSO-treated), and the activation of the downstream PI3K/AKT signaling pathway was also significantly inhibited. This indicated that corylin inhibits GAS6 expression and downstream signaling pathway activation in HSCs, which subsequently inhibits the expression of ECM proteins, such as α-SMA and collagen (Figure 5).

### 2.6. Corylin Inhibited the Expression of MMP Inhibitors, TIMP-1 and TIMP-2, in HSCs

**Figure 6:**
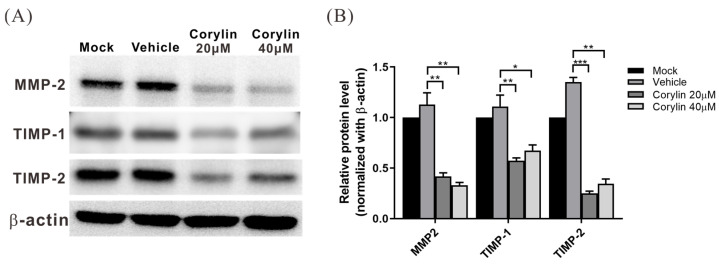
Corylin inhibits TGF-β-induced TIMP-1 and TIMP-2 expression in HHSteC cells. (**A**) Western blotting results of cell lysates of HHSteC cells treated with different concentrations of corylin or vehicle for 2 h and then treated with 4 ng/mL TGF-β to induce an inflammatory response for 24 h; to analyze the expression of MMP-2, TIMP-1, and TIMP-2. Quantitative results are shown in (**B**). All data are expressed as the mean ± standard deviations of three independent experiments. *p* < 0.05 (*), *p* < 0.01 (**), *p* < 0.001 (***). TIMP-1, tissue inhibitor of metalloproteinase 1; MMP-2, matrix metalloproteinase 2.

In addition to ECM, HSCs express matrix metalloproteinase (MMP)-2 and MMP-9 along with their inhibitors, tissue inhibitor of metalloproteinase (TIMP)-1 and TIMP-2, to regulate ECM breakdown. To evaluate the effects of corylin on the expression of these proteins, HHSteC cells were treated with corylin or vehicle for 2 h, followed by TGF-β treatment to stimulate cell activation. Cell lysates were collected after 24 h for Western blotting to analyze the expression of MMP-2, TIMP-1, and TIMP-2. The results showed that TIMP-1 and TIMP-2 expression in the corylin-treated group was significantly lower than that in the control group. In contrast, there was a minor decrease in the expression of MMP-2 (Figure 6), indicating that corylin may upregulate the activity of MMP2 by inhibiting the expression of TIMP1 and TIMP2, thereby accelerating ECM breakdown.

### 2.7. Corylin Treatment Promoted HSC Apoptosis

**Figure 7:**
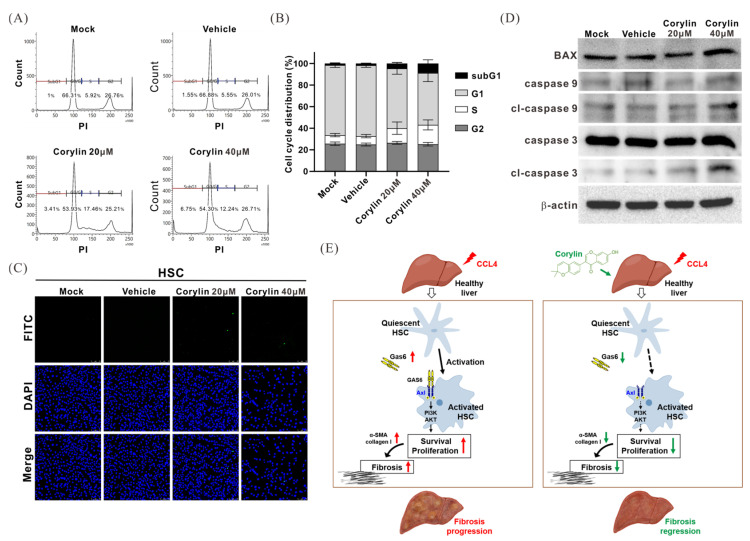
Effects of corylin on apoptosis in HHSteC cells. The apoptotic cell rate and cell cycle status according to (**A**) flow cytometry and (**C**) TUNEL assays of cells incubated with a vehicle (DMSO) and different concentrations of corylin (20 and 40 µM) for 48 h. (**B**) Quantitative results of flow cytometry. Error bars present the mean ± standard deviation from three independent experiments. (**D**) Effects of corylin on apoptosis-related protein expression according to Western blotting analysis. b-actin is the internal control. (**E**) Schematic representation summarizing the anti-liver fibrosis mechanisms of corylin. TUNEL, terminal deoxynucleotidyl transferase dUTP nick-end labeling. Red up arrow indicates up-regulation, and green down arrow means down-regulation.

To further evaluate the effects of corylin on HSC physiology, HHSteC cells were treated with different concentrations of corylin for 48 h. The cells were harvested and subjected to flow cytometry and terminal deoxynucleotidyl transferase dUTP nick-end labeling (TUNEL) assay analysis to assess the apoptosis and cell cycle statuses. Compared to those in the control group, corylin-treated cells were mostly arrested in the S phase, and the number of cells in the sub-G1 phase were significantly increased, indicating that corylin inhibits cell cycle progression and induces apoptosis (Figure 7A,B). TUNEL assay analysis also showed that the number of apoptotic cells in the corylin-treated group increased compared to that in the control group (Figure 7C). Furthermore, Western blotting showed that the levels of cleaved caspase 3 and caspase 9 were significantly increased in corylin-treated HHSteC cells, indicating that corylin promotes HSC apoptosis (Figure 7D).

## 3. Discussion

Chronic hepatitis leads to liver fibrosis and cirrhosis, which are risk factors for liver cancer. However, the early suppression of factors that cause liver injury and inflammation along with the administration of anti-inflammatory drugs can help reverse the progression of liver fibrosis. In this study, we found that corylin, a flavonoid extracted from the fruits of *P. corylifolia*, exhibited anti-inflammatory activity and inhibited the macrophage-mediated secretion of pro-inflammatory cytokines, such as IL-1β, IL-6, and TNF-α. Corylin also inhibited the expression of GAS6 and the downstream activation of the PI3K/AKT signaling pathway in HSCs, thereby inhibiting HSC activation and the expression of ECM proteins, including α-SMA and collagen. Moreover, corylin treatment alleviated the symptoms of CCl_4_-induced liver fibrosis in a mouse model. These findings suggest that corylin has the potential to be used in the treatment of hepatitis and liver fibrosis. To the best of our knowledge, this is the first study to demonstrate that corylin inhibits GAS6 expression and subsequently inhibits HSC activation to alleviate the symptoms of liver fibrosis in mice (Figure 7E).

RTK AXL is expressed in most tissues and is involved in the regulation of diverse cellular physiological processes, including growth, survival, differentiation, adhesion, and migration. GAS6/AXL signaling is also involved in the regulation of macrophage polarization and the inflammatory response [42,43]. The downstream signaling pathway of AXL activation induces macrophage polarization into the M2 type, which subsequently downregulates pro-inflammatory cytokines, such as IL-6, TNF, type-I IFNs, and IL-12. Most tumor cells activate the downstream signaling pathway of TAM receptors by secreting GAS6, which subsequently inhibits macrophage activation and pro-inflammatory cytokine expression, creating an immune-tolerant environment around the tumor, which helps cancer cells survive during the immune response [44,45]. In the present study, we also analyzed the effect of corylin on the expression of GAS6 and AXL in THP1 cells. However, there was no significant change in their expression, indicating that corylin does not inhibit macrophage activation and pro-inflammatory cytokine expression by regulating the AXL signaling pathway. This also suggests that corylin is cell-specific in its regulation of physiological processes.

GAS6 supports hematopoietic stem cell growth and promotes fibroblast and endothelial cell survival [46,47]. In addition, GAS6/AXL signaling induces the accumulation of mesangial cells in kidney fibrosis [48], vascular smooth muscle cells in response to intimal vascular injury [49], and cardiac fibroblasts during the wound-healing process [50], thereby suggesting that GAS6 plays an important role in tissue fibrosis. Furthermore, GAS6 modulates HSC and HSC/myofibroblastoma survival during liver repair after acute injury [51]. The results of our study showed that corylin promoted HSC apoptosis, and part of this effect may have been achieved by inhibiting GAS6 expression.

Activated HSCs not only regulate the expression of ECM proteins, but also regulate the expression and secretion of MMP-2 and MMP-9 along with their inhibitors, TIMP-1 and TIMP-2, to regulate ECM breakdown [52,53]. During fibrogenesis, this equilibrium is disturbed, and the expression of TIMPs and MMPs is increased leading to an excess of TIMPs and subsequent matrix degradation. In the present study, we found that corylin inhibited the expression of ECM proteins, including α-SMA and collagen, in HSCs and also inhibited the expression of MMP inhibitors, TIMP-1 and TIMP-2, which may upregulate MMP-2 activity to accelerate ECM breakdown. In addition, the increased expression of MMP-2, MMP-9, and TIMP-1 has been regarded as an indicator of HSC activation [54]. The corylin-mediated inhibition of these proteins also indicates the inhibitory effect of corylin on HSC activation. Furthermore, corylin promoted apoptosis in HSCs. These findings showed that corylin simultaneously regulated multiple pathways to inhibit the progression of liver fibrosis. In addition to HSCs, MMP-2 and MMP-9 are expressed in most inflammatory cells, such as lymphocytes, neutrophils, macrophages, and Kupffer cells [55]. Thus, the effects of corylin on the expression of TIMP-1 and MMP-9 in macrophages and Kupffer cells should be further studied to clarify the mechanism by which corylin inhibits liver fibrosis.

In this study, we demonstrated the anti-inflammatory activity of corylin, an extract of *P. corylifolia*, and its efficacy in treating liver fibrosis. Corylin has no obvious physiological toxicity and thus has great potential to be used as an adjuvant in clinical treatment. We have clarified the anti-inflammatory molecular mechanism of corylin and its potential for clinical application.

## 4. Materials and Methods

### 4.1. Cell Lines

The human monocyte cell line THP1, and mouse macrophage cell line RAW 264.7 were purchased from the American Type Culture Collection (Manassas, VA, USA). The aforementioned cells were cultured in Dulbecco’s modified Eagle’s medium containing 10% fetal bovine serum at 37 °C in a 5% CO_2_ atmosphere. The human HSC cell line HHSteC was purchased from the ScienCell Research Laboratories (Carlsbad, CA, USA) and cultured using Stellate Cell Medium.

### 4.2. Materials and Reagents

Primary antibodies against AXL, phosphorylated (phospho)-AXL, GAS6, phospho-PI3K, PI3K, phospho-AKT, AKT, IL-1β, IL-6, MMP-2, MMP-9, TIMP-1, TIMP2, cleaved caspase-3, caspase-3, cleaved caspase-9, and caspase-9 were purchased from Genetex (Irvine, CA, USA), ABclonal (Woburn, MA, USA), and Cell Signaling Technology (Beverly, MA, USA). Secondary antibodies were purchased from Santa Cruz Biotechnology (Santa Cruz, CA, USA). Pre-stained protein marker and TOOLSmart RNA extractor were purchased from BIOTOOLS (Taipei, Taiwan). Corylin powder (purity above 98% as measured by high-performance liquid chromatography) was purchased from Shanghai BS Bio-Tech (Shanghai, China).

### 4.3. Western Blot Analysis

Cells treated with different concentrations of corylin for 24 and 48 h were harvested and washed twice with phosphate-buffered saline (PBS) and then lysed in 200 μL of radioimmunoprecipitation assay lysis buffer (BIOTOOLS) containing a protease inhibitor. Protein (30 μg) from the supernatant was loaded onto a sodium dodecyl sulfate polyacrylamide gel, followed by Western blot analysis to detect the levels of target proteins. Detailed information of antibodies used in the experiments is shown Table S1. The immuno-reactive bands were revealed using an enhanced chemiluminescence system (NEN Life Science Products, Boston, MA, USA) and detected using UVP ChemStudio Imaging Systems (Analytik Jena, Upland, CA, USA). The intensity of each band was quantified using ImageQuant 5.2 (GE Healthcare, Piscataway, NJ, USA).

### 4.4. Enzyme-Linked Immunosorbent Assay

THP-1 and RAW 264.7 cells were treated with different concentrations of corylin for 2 h followed by treatment with lipopolysaccharide (LPS) for 24 h to induce an inflammatory response. The protein levels of IL-1β, IL-6, and TNF-α in the culture medium were measured using ELISA kits (BioLegend, San Diego, CA, USA) according to the manufacturer’s instructions.

### 4.5. Flow Cytometry

HHSteC cells were treated with DMSO, 20 µM corylin, and 40 µM corylin for 24 h, followed by trypsinization and then washed twice and incubated in PBS containing 0.12% Triton X-100, 0.12 mM ethylenediaminetetraacetic acid, and 100 mg/mL ribonuclease A. Propidium iodide (50 μg/mL) was then added to each sample for 20 min at 4 °C. Cell cycle distribution was analyzed using flow cytometry (Beckman Coulter Epics Elite, Beckman Coulter, Brea, CA, USA).

### 4.6. Terminal Deoxynucleotidyl Transferase dUTP Nick-End Labeling Assay

The apoptosis status of HHSteC cells was determined using a DeadEnd^TM^ Fluorometric TUNEL Assay Kit (Promega, Madison, WI, USA) according to the manufacturer’s protocol. Briefly, HHSteC cells were treated with DMSO, 20 µM corylin, or 40 µM corylin for 24 h. The cells were then subjected to a TUNEL assay. The cells were counted using a microscope (magnification, ×100). Cells in five different microscopic fields/dishes were analyzed for each experiment.

### 4.7. Mice

Male BALB/c mice (age, 6–8 weeks; National Laboratory animal center, Taipei, Taiwan) were housed under pathogen-free conditions with a 12 h light/12 h dark schedule and fed autoclaved standard chow and water. All animal experiments were approved by the Institutional Animal Care and Use Committee (IACUC) at Chang-Gung memorial Hospital (IACUC approval no.: 2019032009, approval date: 2019/6/11).

### 4.8. CCl_4_-Induced Liver Fibrosis Mouse Model

A total of 20 mice were randomly assigned to three groups: negative control (control, *n* = 6), CCl_4_ treatment + DMSO (vehicle, *n* = 7), and CCl_4_ treatment + corylin (30 mg/kg, *n* = 7). CCl_4_ was liquefied in olive oil to obtain a 10% CCl_4_ solution that was injected intraperitoneally into mice (0.5 μL/g body weight) twice a week for six weeks. At the beginning of the second week, mice were intraperitoneally injected with 100 µL of corylin (at a dose of 30 mg/kg of body weight) or an equal volume of DMSO as a control for 3 d per week. Negative control mice were treated with olive oil alone. At the endpoint, blood samples were collected to measure the levels of serum AST and ALT. Liver tissues were collected for further assays such as histology and Western blotting.

### 4.9. Masson’s Trichrome Staining

Masson’s trichrome staining was performed at the Chang Gung Memorial Hospital Department of Anatomic Pathology as follows: First, 5 μm thick formalin-fixed paraffin-embedded (FFPE) sections were deparaffinized and hydrated in distilled water. Subsequently, Bouin’s fixative was used as a mordant for 1 h at 56 °C. The FFPE sections were cooled and washed in running water until the yellow color disappeared. The samples were stained in Weigert’s hematoxylin stain for 10 min, thoroughly washed in tap water for 10 min, stained in an acid fuchsin solution for 15 min, and then rinsed in distilled water for 3 min. After rinsing, the slides were treated with phosphomolybdic acid solution for 10 min and rinsed in distilled water for 10 min. Finally, the slides were stained with a light-green solution for 2 min and rinsed in distilled water. After thorough dehydration using alcohol, the slides were mounted, and coverslips were placed onto them.

### 4.10. Immunohistochemistry

Mouse liver tissues were fixed in formalin and embedded in paraffin, and 2 μm thick consecutive sections were cut and subjected to immunohistochemical staining using a BOND III autostainer (Leica Biosystems, Wetzlar, Germany) as described previously [35].

### 4.11. Data Analysis

The quantitative real-time polymerase chain reaction and Western blot data were recorded as continuous variants and analyzed using the Student’s *t*-test. All statistical analyses were performed using SPSS 16.0 (IBM, Armonk, NY, USA) and Excel 2007. All statistical tests were two-sided, and *p* values < 0.05 (*), <0.01 (**), and <0.001 (***) were considered significant.

## Data Availability

All data analyzed during this study are included in this article. Further inquiries can be directed towards the corresponding author.

## Supplementary Materials

The following supporting information can be downloaded at: https://www.mdpi.com/article/10.3390/ijms242316936/s1.
