# Supplementary material for: Corylin Attenuates CCl4-Induced Liver Fibrosis in Mice by Regulating the GAS6/AXL Signaling Pathway in Hepatic Stellate Cells
Source: Int J Mol Sci. 2023 Nov 29;24(23):16936. doi: 10.3390/ijms242316936 (PMC10707553; doi:10.3390/ijms242316936)
Supplement: Supplementary file 1 [file ijms-24-16936-s001.zip › ijms-2727112-supplementary.pdf]

Table S1. Antibodies used in the experiments.

**Antibody**	**Source information** (vendor, cat#)	**Dosage**	**Use**
pERK	Cell signaling #4370	1:1000 dilution	WB
ERK	Cell signaling #4695	1:1000 dilution	WB
pJNK	Cell signaling #4668	1:1000 dilution	WB
JNK	Cell signaling #9252	1:1000 dilution	WB
pP38	Cell signaling #4511	1:1000 dilution	WB
P38	Cell signaling #8690	1:1000 dilution	WB
GAS6	Cell signaling #67202	1:1000 dilution	WB
pAXL	Cell signaling #5724	1:1000 dilution	WB
AXL	Cell signaling #8661	1:1000 dilution	WB
pPI3K	Cell signaling #4228	1:1000 dilution	WB
PI3K	Cell signaling #4292	1:1000 dilution	WB
pAKT	Cell signaling #9271	1:1000 dilution	WB
AKT	Cell signaling #9272	1:1000 dilution	WB
α-SMA	Cell signaling #19245	1:1000 dilution	WB
	Cell signaling #19245	1:200 dilution	IHC
COL1A1	Cell signaling #72026	1:1000 dilution	WB
	Cell signaling #72026	1:200 dilution	IHC
MMP-2	ABclonal #A19080	1:1000 dilution	WB
TIMP-1	Cell signaling #8946	1:1000 dilution	WB
TIMP-2	Cell signaling #5738	1:1000 dilution	WB
cl- caspase-9	Cell signaling #9505	1:1000 dilution	WB
Caspase-9	Cell signaling #9502	1:1000 dilution	WB
cl-caspase-3	Cell signaling #9661	1:1000 dilution	WB
caspase-3	Cell signaling #9662	1:1000 dilution	WB
BAX	Cell signaling #2772	1:1000 dilution	WB
β-Actin	GENETEX # GTX109639	1:1000 dilution	WB
